# Immune Checkpoint Inhibition in Patients with Brain Metastases from Non-Small-Cell Lung Cancer: Emerging Mechanisms and Personalized Clinical Strategies

**DOI:** 10.3390/ijms26178624

**Published:** 2025-09-04

**Authors:** Nicola J. Nasser, Kunal K. Sindhu, Loor Nasser, Zahra Shafaee, Joshua Li, Lucas Resende Salgado, Baoqing Li

**Affiliations:** 1 The Mount Sinai Hospital, Icahn School of Medicine at Mount Sinai, New York, NY 10029, USA; kunal.sindhu@mountsinai.org (K.K.S.); lucas.resendesalgado@mountsinai.org (L.R.S.); 2 Riverdale Kingsbridge Academy, Bronx, NY 10463, USA; nassloor@gmail.com; 3 NYC Health + Hospitals/Elmhurst, Queens, NY 11373, USA; shafaeez1@nychhc.org; 4 Department of Chemistry, University of Rochester, Rochester, NY 14627, USA; jli221@u.rochester.edu; 5 Weill Cornell Medicine, New York, NY 10065, USA; bal9018@med.cornell.edu

**Keywords:** brain metastases, non-small-cell lung cancer, immune checkpoint inhibitors, intracranial response, blood–brain barrier, tumor microenvironment, immunotherapy

## Abstract

Brain metastases are a significant complication of non-small-cell lung cancer (NSCLC), contributing to high morbidity and mortality rates. The introduction of immune checkpoint inhibitors (ICIs) has opened new therapeutic avenues for patients with NSCLC, including those with brain metastases. However, the distinct microenvironment of the brain presents unique challenges to the effectiveness of these treatments. This review examines the mechanisms by which ICIs impact brain metastases from NSCLC, with particular focus on immune cell trafficking across the blood–brain barrier (BBB), tumor microenvironment modulation, and transcriptomic evolution of brain-tropic tumor clones. Unlike prior reviews, we integrate emerging data from single-cell and spatial transcriptomic studies, BBB disruption mechanisms, and the tumor-supportive role of brain-resident glia. We also critically evaluate key clinical trials and real-world evidence, highlighting differences in ICI efficacy across patient subgroups and therapeutic contexts. Additionally, we address the evolving role of surgical resection, stereotactic radiosurgery, and cerebrospinal-fluid-based biomarkers in optimizing ICI-based treatment strategies. This synthesis provides a comprehensive, mechanistic, and clinically relevant framework for improving outcomes in patients with NSCLC brain metastases treated with immunotherapy.

## 1. Introduction

Brain metastases occur in approximately 20–40% of patients with non-small-cell lung cancer (NSCLC) and significantly impact patient outcomes [1,2,3]. The prognosis for patients with brain metastases depends heavily on the number and size of metastases and the overall systemic burden of disease. Patients with a single brain metastasis and no other metastatic disease can achieve long-term disease-free survival through aggressive treatment strategies targeting both the primary lung tumor and the metastatic brain lesion [4]. These strategies may include concomitant chemotherapy and radiation therapy or surgical resection of the primary tumor, coupled with stereotactic radiosurgery (SRS) or surgical resection of the brain metastasis.

However, the prognosis becomes progressively worse for patients with two to three brain metastases and even more so for those with four or more brain metastases [5]. For these patients, the prognosis remains poor despite advancements in systemic therapies because of the limited ability of many treatments to penetrate the central nervous system (CNS) and effectively target tumor cells. Additionally, the potential presence of additional lesions that are below the MRI detection threshold can limit the ability of a course of SRS to achieve extended intervals of intracranial progression-free survival when targeting multiple lesions in the brain.

Immune checkpoint inhibitors (ICIs), such as those targeting PD-1, PD-L1, and CTLA-4, have emerged as promising therapies for locally advanced and metastatic cancers [6,7,8,9,10,11,12,13,14], including NSCLC [15,16,17,18,19,20]. These therapies have shown potential in enhancing the immune system’s ability to target and eliminate cancer cells, including those in the CNS. However, the unique immunological and physiological landscape of the brain presents specific challenges to the efficacy of ICIs in treating brain metastases [21]. This review examines the mechanisms by which ICIs affect brain metastases from NSCLC and discusses the clinical implications of these findings. Specifically, we synthesize recent advances in blood–brain barrier biology, tumor microenvironment modulation, and single-cell transcriptomic profiling to explore why certain metastatic subtypes respond differently to ICI therapy. We incorporate real-world trial data and highlight emerging therapeutic strategies—such as combining ICIs with surgical resection, stereotactic radiosurgery, or cerebrospinal-fluid-based monitoring—to propose a more personalized framework for managing brain metastases in NSCLC. This narrative, mechanism-focused review prioritizes original intracranial data when available, contrasts consistent and conflicting findings across studies and tumor types, and identifies unresolved questions to guide future trial design.

## 2. Mechanisms of ICI Action in Brain Metastases

The effectiveness of ICIs in treating brain metastases from NSCLC depends on their ability to activate T cells both systemically and within the CNS, despite the immune-privileged nature of the brain and the presence of the blood–brain barrier (BBB) [21,22,23,24]. Recent studies specific to NSCLC brain metastases have confirmed that ICIs can show CNS activity even in the presence of limited BBB penetration [21,24].

### 2.1. Immune Checkpoint Inhibition and T-Cell Activation

#### 2.1.1. Activation of Peripheral T Cells and Suppression of Extracranial Tumor Cells

**Figure 1:**
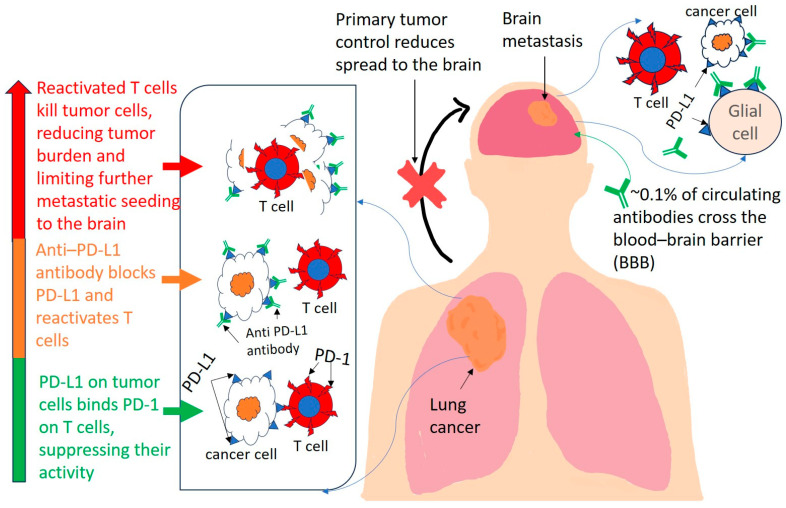
Schematic illustration of a patient with non-small-cell lung cancer (NSCLC) and brain metastasis. The primary tumor is located in the right lung, with a metastatic lesion in the brain. Tumor cells express PD-L1, while infiltrating T cells express PD-1. An immune checkpoint inhibitor (anti-PD-L1 antibody in this illustration) blocks PD-L1, promoting T-cell activation and tumor control. The inhibited arrow indicates suppressed tumor growth and potentially reduced further metastatic spread to the brain. Although only ~0.1% of circulating antibodies cross the blood–brain barrier (BBB), this limited penetration may still activate brain-resident or infiltrating T cells, contributing to immune-mediated control of brain metastases. Microglia and astrocytes—two major resident glial cell types—can upregulate PD-L1 in response to inflammatory stimuli or tumor presence, creating an immunosuppressive microenvironment. Anti-PD-L1 therapy may act not only by reactivating T cells but by reprogramming brain-resident glia to reduce PD-L1 expression, thereby shifting the local milieu toward one that supports cytotoxic immune responses against metastatic cancer cells in the brain.

ICIs work by blocking inhibitory immune checkpoints, such as PD-1/PD-L1 and CTLA-4, which restores the ability of T cells to detect and destroy tumor cells [25]. In patients with NSCLC, this peripheral activation leads to the suppression of systemic tumor burden, reducing the likelihood of new metastatic seeding in the brain (Figure 1). Therefore, part of the intracranial benefit of ICIs may derive from their impact on extracranial disease control.

In addition to PD-1, PD-L1, and CTLA-4, other inhibitory receptors—including T cell immunoglobulin and mucin-domain containing-3 (TIM-3) and lymphocyte activation gene-3 (LAG-3)—are increasingly recognized as markers and mediators of T-cell dysfunction within the tumor microenvironment [26,27,28]. TIM-3 expression on CD8^+^ T cells correlates with impaired cytotoxic responses, while LAG-3 can synergize with PD-1 to blunt antitumor immunity [26,27,28]. Although clinical targeting of these pathways in NSCLC brain metastases remains limited, their upregulation in intracranial lesions suggests potential combinatorial ICI strategies.

#### 2.1.2. TIM-3, LAG-3, and Emerging Checkpoints in Brain Metastases

Beyond PD-1/PD-L1, supplementary checkpoint molecules such as TIM-3 and LAG-3 are increasingly implicated in the immune landscape of NSCLC brain metastases. Lu et al. [29] demonstrated that although TIM-3 and LAG-3 expression on CD3^+^ T cells was lower in NSCLC brain metastases compared with primary lung tumors, their expression was nevertheless associated with improved overall survival. This suggests that TIM-3 and LAG-3 can mark clonally expanded, tumor-reactive T cells in inflamed tumors rather than exclusively indicating terminal exhaustion. Xiao et al. [30] developed an immune scoring system (GYMS) from RNA-seq and IHC analysis of 70 lung adenocarcinoma brain metastases. High-GYMS tumors exhibited an inflamed immune landscape—characterized by elevated immune cell infiltration, enhanced antigen-presentation pathways, and increased expression of multiple immune checkpoint genes, including LAG3, TIM-3, PD-1, and CTLA-4—and were associated with improved overall survival. These results underscore the prognostic and mechanistic relevance of TIM-3 and LAG-3 within the brain metastatic microenvironment and reinforce the rationale for evaluating combinatorial checkpoint strategies with CNS-specific endpoints.

#### 2.1.3. Activation of Brain-Resident or Infiltrating T Cells

Although the normal brain contains only low numbers of resident T cells, particularly in the meninges, perivascular spaces, and choroid plexus, disease states such as brain metastases promote immune cell infiltration into the parenchyma. Despite the limited ability of ICIs to cross the BBB [31]—estimated at ~0.1% of circulating antibody levels [32]—this small amount may be sufficient to activate local or infiltrating T cells, leading to localized immune responses against metastatic lesions. In addition, microglia and astrocytes—two major resident glial cell types in the brain—can upregulate immune checkpoint ligands such as PD-L1 in response to inflammatory stimuli or tumor presence, thereby contributing to an immunosuppressive microenvironment [33] (Figure 1). ICIs may function not only by reactivating T cells but by reprogramming brain-resident glia to reduce PD-L1 expression, shifting the local milieu toward one that supports cytotoxic immune responses. Inflammatory signals and BBB disruption further support this immune activation within the brain.

### 2.2. The Blood–Brain Barrier and Immune Cell Trafficking

**Figure 2:**
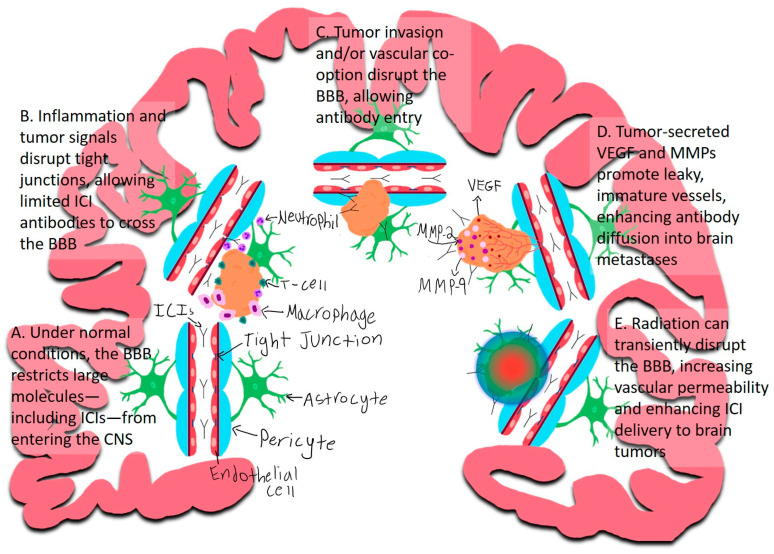
Schematic representation of potential mechanisms by which immune checkpoint inhibitor (ICI) antibodies reach the brain in patients with NSCLC. The blood–brain barrier (BBB) is composed primarily of tightly connected endothelial cells supported by pericytes and astrocytic end-feet. Tight junctions between endothelial cells restrict paracellular transport, limiting the entry of circulating molecules and immune cells. Pericytes wrap around endothelial cells, regulating vascular stability and permeability, while astrocyte end-feet encase the vasculature, providing metabolic support and further reinforcing BBB integrity. This highly selective barrier protects the central nervous system from toxins and pathogens while also limiting the penetration of therapeutic agents, including ICIs. (A) Under normal physiological conditions, the BBB limits the entry of large molecules such as ICI antibodies into the CNS. (B) Inflammatory signals associated with systemic immune activation or local tumor-associated inflammation may disrupt tight junctions and increase BBB permeability, allowing limited antibody access. (C) Direct tumor invasion into, or vascular co-option of, brain vasculature can compromise the integrity of the BBB. Invasion physically disrupts endothelial junctions, while co-option involves tumor cells migrating along the outer surface of vessels, both leading to localized increases in vascular permeability and facilitating antibody penetration. (D) Tumor-secreted factors such as vascular endothelial growth factor (VEGF) and matrix metalloproteinases (MMPs) can promote abnormal angiogenesis, resulting in leaky and immature blood vessels within metastatic lesions, further enhancing antibody diffusion into the brain parenchyma. (E) Radiation therapy directed at brain metastases can transiently disrupt the BBB by affecting nearby blood vessels, increasing vascular permeability and potentially improving ICI delivery to CNS tumors.

The blood–brain barrier (BBB) is a highly selective neurovascular interface composed of endothelial cells, tight junctions, pericytes, and astrocyte end-feet, which collectively restrict the entry of immune cells and large molecules, such as monoclonal antibodies, into the brain parenchyma (Figure 2) [31,34].

However, in the setting of brain metastases from NSCLC, the BBB can become transiently permeable through multiple mechanisms (Figure 2):

#### 2.2.1. Inflammation

Metastatic lesions can induce localized inflammation within the brain microenvironment. Infiltrating immune cells—such as neutrophils and monocytes—exacerbate blood–brain barrier (BBB) disruption by releasing reactive oxygen species, cytokines, and proteolytic enzymes. These inflammatory mediators compromise endothelial integrity and tight junctions, leading to increased BBB permeability and facilitating the entry of immune checkpoint inhibitors into the central nervous system [35,36,37,38].

#### 2.2.2. Direct Tumor Invasion and/or Vascular Co-Option

Tumor cells can directly invade intracranial blood vessels or utilize vascular co-option, whereby they migrate along the abluminal surface of existing vessels. Both processes can physically disrupt endothelial integrity and promote localized vascular permeability, thereby compromising the blood–brain barrier and potentially facilitating the entry of immune checkpoint inhibitors [39,40,41,42].

#### 2.2.3. Tumor-Induced Angiogenesis and BBB Disruption

Tumor cells contribute to BBB disruption through secretion of propermeability and matrix-degrading factors. Vascular endothelial growth factor (VEGF) downregulates tight junction proteins such as claudin-5 and occludin in endothelial cells, weakening barrier integrity [43,44], while matrix metalloproteinases (MMP-2, MMP-9) degrade the vascular basement membrane and extracellular matrix [45,46]. Hypoxic conditions in the tumor microenvironment further stimulate VEGF production, driving abnormal angiogenesis [45,46,47]. The resulting vasculature is immature, with poor pericyte and astrocyte support, leading to increased leakiness and impaired BBB function [48,49,50,51]. These pathways are under investigation as therapeutic targets, and dual inhibition strategies (e.g., anti-VEGF plus ICI) are being explored in preclinical models to enhance CNS drug delivery [52,53].

#### 2.2.4. Radiation-Induced BBB Disruption

**Figure 3:**
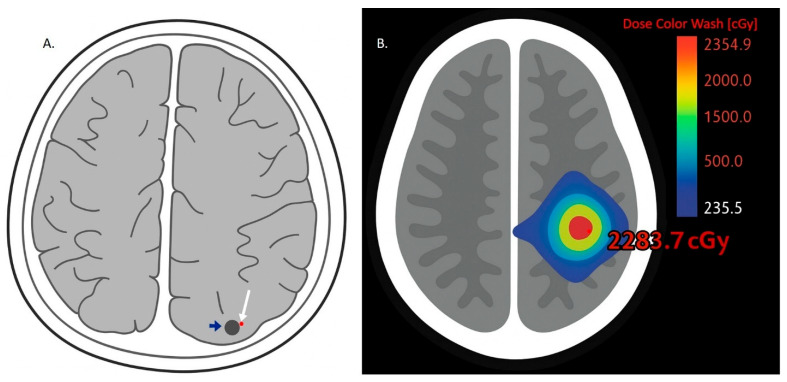
(**A**) Schematic illustration based on an axial postcontrast T1-weighted MRI, showing a small lesion in the left occipital lobe (blue arrow) representing a brain metastasis from non-small-cell lung cancer (NSCLC). The lesion (blue arrow) abuts a blood vessel (white arrow), with no significant surrounding edema. (**B**) Schematic illustration of a stereotactic radiosurgery (SRS) treatment plan for a brain metastasis in NSCLC. A color wash overlay demonstrates the radiation dose distribution in centigrays (cGy), with a central maximum dose of 2283.7 cGy and a prescription dose of 2000 cGy in a single fraction. The gradient (red to blue) illustrates decreasing dose from the tumor center to adjacent brain tissue and vessels, highlighting potential effects on the blood–brain barrier.

Radiation therapy (RT), including whole-brain radiation therapy (WBRT) and stereotactic radiosurgery (SRS), is a cornerstone in the nonsurgical management of brain metastases (Figure 3) [54,55]. Emerging evidence indicates that RT can transiently increase BBB permeability through multiple mechanisms [56,57,58]. Radiation-induced damage to endothelial cells leads to downregulation of tight junction proteins, alterations in membrane transporter expression, and endothelial apoptosis, collectively compromising BBB integrity [58,59]. In addition, RT promotes the release of proinflammatory cytokines, further contributing to increased vascular permeability [60,61]. These changes—including enhanced BBB permeability—may facilitate the penetration of therapeutic agents, including chemotherapeutics, ICIs, and immune cells, into the CNS (Figure 2). However, the extent and duration of BBB disruption following RT are variable and may depend on factors such as radiation dose, fractionation schedule, and individual patient characteristics [62].

Once within the brain, activated T cells may engage tumor cells directly, contributing to therapeutic efficacy. Thus, trafficking across the BBB represents not only a critical bottleneck but a potential therapeutic window for immune intervention [31]. While ICIs can activate T cells systemically [63,64] and within the brain [21,22,24], their intracranial efficacy is ultimately shaped by the tumor microenvironment, which can either support or suppress immune-mediated tumor destruction. This is discussed in the following section.

## 3. The Tumor Microenvironment in Patients with Brain Metastases

The TME in the brain is composed of both resident glial cells—such as astrocytes [65] and microglia [66,67]—and infiltrating immune cells that interact with metastatic tumor cells. These interactions are dynamic and can either promote or inhibit antitumor immune responses, thus playing a pivotal role in determining the success of ICIs in the CNS (Figure 1).

In addition to these environmental factors, tumor cells themselves may undergo genetic adaptations that promote colonization in the brain. For instance, Wang et al. [68] used single-cell RNA sequencing to show that lung cancer cells metastasizing to the brain exhibit unique transcriptomic profiles and evolutionary patterns, supporting the idea that certain molecular subtypes are better equipped to survive in the CNS.

More recently, Xiao et al. [69] combined single-cell RNA sequencing with spatial transcriptomics to identify a distinct subpopulation of epithelial cells in lung cancer brain metastases, termed early metastatic epithelial cell clusters (EMEC). These cells were enriched in oxidative phosphorylation and coagulation pathways, engaged in extensive ligand–receptor signaling with immune cells, and consistently associated with poor prognosis. Spatial analyses revealed a progressive shift of EMEC from the tumor periphery toward its central regions during invasion, suggesting a dynamic adaptation to the brain microenvironment that may influence metastatic progression and treatment response. Complementing these findings, Shi et al. [70] used integrative single-cell and bulk transcriptomics in early-stage lung adenocarcinoma to identify molecular drivers of tumor aggressiveness, notably KRT8 and PERP, which promote epithelial cell proliferation and migration. The involvement of KRT8-positive transitional epithelial states in both early tumorigenesis and brain colonization underscores potential shared molecular programs that could be exploited for biomarker development and therapeutic targeting in brain metastases.

### 3.1. PD-L1 Expression in the CNS

PD-L1 expression can be upregulated in the brain’s microenvironment, including on astrocytes and other resident cells, particularly under inflammatory conditions or in the presence of tumors. This expression can inhibit T-cell activity by engaging with PD-1 on T-cells, contributing to an immunosuppressive environment that supports tumor survival and growth (Figure 1). Understanding how ICIs can modulate this interaction is critical for improving treatment outcomes in patients with brain metastases.

Within brain metastases, coexpression of checkpoint molecules such as PD-1, TIM-3, and LAG-3 on tumor-infiltrating lymphocytes is common and in many contexts reflects a state of terminal exhaustion often unresponsive to PD-1/PD-L1 blockade alone. In preclinical CNS tumor models, TIM-3 blockade has been shown to partially restore T-cell function. In addition, ICIs may alter the tumor microenvironment not only by reinvigorating T cells but by reprogramming brain-resident glial cells toward a more inflammatory, antitumor phenotype [26,27,28]. This includes the recruitment of activated CD8^+^ T cells and the shift of astrocytes and microglia from a tumor-supportive to a tumor-suppressive state. These microenvironmental changes are essential for sustaining an effective intracranial response and may explain the heterogeneity of outcomes observed in patients treated with ICIs [67,71,72].

In brain metastases, activated astrocytes and microglia can upregulate PD-L1 in response to cytokines such as IFN-γ [66,73,74,75]. This glial PD-L1 interacts with PD-1 on infiltrating CD8^+^ T cells, dampening local immune responses and promoting T-cell exhaustion. Thus, even in cases where tumor cells express low PD-L1, the glial compartment may create an immunosuppressive niche that reduces ICI efficacy. While tumor PD-L1 expression is a key biomarker for ICI response, PD-L1 expressed by glial cells may independently suppress T-cell activation and contribute to CNS immune escape, regardless of antibody availability or tumor expression levels [33]. This mechanism may explain some cases of intracranial ICI resistance and underscores the need to consider the nontumor cellular context when evaluating checkpoint inhibition in the CNS [33,66,75].

### 3.2. Tumor-Associated Macrophages and Dendritic Cells

Beyond glial cells, other myeloid populations—including tumor-associated macrophages (TAMs) and dendritic cells (DCs)—play pivotal roles in shaping the brain metastatic niche. Myeloid infiltration is now recognized as a common feature across brain metastases of diverse primary tumors [76], with TAMs arising from both resident perivascular macrophages and infiltrating monocytes. Phenotypically, TAMs in the CNS microenvironment exhibit a predominantly immunosuppressive M2-like profile, producing IL-10, TGF-β, and VEGF, which collectively blunt cytotoxic T cell responses and promote angiogenesis [76,77,78,79]. Importantly, experimental inhibition of the anti-inflammatory TAM phenotype—via genetic deletion of KLF4 or pharmacologic blockade of CSF-1R/STAT6 signaling—significantly reduced brain metastatic burden in murine breast cancer models [80].

Dendritic cells, although less abundant, are central regulators of antigen presentation and T cell priming in the CNS [81,82,83,84]. Preclinical work suggests that brain metastases harbor tolerogenic DC subsets with impaired costimulatory molecule expression, limiting their ability to generate robust antitumor immunity [82,85,86,87]. Moreover, interactions between DCs and T cells are spatially constrained within perivascular niches, where tumor-derived cytokines such as CSF-1 and IL-6 further impair antigen presentation [88,89,90].

Together, these myeloid populations contribute to an immunosuppressive intracranial tumor microenvironment that is mechanistically distinct from glial–immune interactions. Importantly, their presence may explain, in part, the limited efficacy of PD-1/PD-L1 blockade alone in brain metastases and underscores the need for therapeutic strategies that simultaneously target myeloid-driven immunoregulation [91].

## 4. Clinical Evidence of ICI Efficacy in Patients with Brain Metastases from NSCLC

Several clinical trials have investigated the efficacy of ICIs in patients with brain metastases from NSCLC. These studies provide insights into how ICIs perform in the unique environment of the brain [92]. Table 1 provides a summary of major clinical trials evaluating immune checkpoint inhibitors (ICIs) in patients with non-small-cell lung cancer (NSCLC) and brain metastases, highlighting intracranial response rates and key findings.

### 4.1. Clinical Trials and Outcomes

- CheckMate 017 [93] and CheckMate 057 Trials [94]:
  ○ CheckMate 017 [93]: This phase III trial evaluated nivolumab in patients with previously treated advanced squamous NSCLC. Patients with treated, stable brain metastases were eligible. The results demonstrated that nivolumab significantly improved overall survival (OS) as compared with docetaxel, with a hazard ratio of 0.59 (95% CI, 0.44 to 0.79). At one year, the overall survival rate was 42% (95% CI, 34% to 50%) with nivolumab as compared with 24% (95% CI, 17% to 31%) with docetaxel. This trial established the efficacy of nivolumab as a second-line therapy for squamous NSCLC [93]. Outcomes in patients with brain metastases were not separately reported.
  ○ CheckMate 057 [94]: This phase III trial focused on patients with previously treated nonsquamous NSCLC. Similar to CheckMate 017, nivolumab showed a significant improvement in overall survival (OS) as compared with docetaxel. The median OS was 12.2 months (95% CI, 9.7 to 15.1 months) with nivolumab, compared with 9.4 months (95% CI, 8.1 to 10.7 months) with docetaxel, with a hazard ratio (HR) of 0.72 (95% CI, 0.59 to 0.89). At 18 months, the rate of overall survival was 39% (95% CI, 34% to 45%) with nivolumab and 23% (95% CI, 19% to 28%) with docetaxel. Patients with stable brain metastases were eligible to participate if they were asymptomatic and did not require corticosteroids [94]. Outcomes in patients with brain metastases were not separately reported.

Combined Analysis [100]: A pooled analysis of the CheckMate 017 and CheckMate 057 trials, with a follow-up time of five years, showed that nivolumab provided consistent survival benefits across various subgroups of patients, including those who had different metastatic sites and lines of prior therapy. This long-term follow-up highlighted the durability of nivolumab’s benefit, suggesting that it is a valuable treatment option for a broad range of heavily pretreated NSCLC patients, potentially including those with stable brain metastases, although further specific data is needed for these subgroups [100].

- CheckMate 063 and Expanded Access Program (EAP) Findings:
  ○ CheckMate 063 [101]: This phase II, single-arm trial evaluated the efficacy and safety of nivolumab in patients with squamous non-small-cell lung cancer (NSCLC) who had progressed after two or more lines of therapy [101]. While the trial provided important early evidence of nivolumab’s efficacy in a heavily pretreated patient population, it included only a small number of patients with brain metastases. Consequently, while CheckMate 063 supports the use of nivolumab in advanced squamous NSCLC, its specific findings related to patients with brain metastases are limited, highlighting the need for further research to better understand its effects in this subgroup.
  ○ Expanded Access Program (EAP) in Italy [99]: The EAP was initiated to provide access to nivolumab for patients with nonsquamous NSCLC, including those with brain metastases, who might not have qualified for clinical trials [99]. Patients with brain metastases were eligible for the EAP if they were asymptomatic, neurologically stable, and either off corticosteroids or on a stable or decreasing dose. The program demonstrated a disease control rate (DCR) of 39% and a median OS of 8.6 months among patients with brain metastases, suggesting that nivolumab can offer clinical benefits even in this challenging patient population with poor prognoses. Although the EAP was not directly started because of CheckMate 063, it was informed by the data from early trials and aimed to provide broader access to nivolumab in a real-world setting [99].
- KEYNOTE-189 Trial [95]: This trial evaluated the combination of pembrolizumab with chemotherapy versus chemotherapy alone in patients with nonsquamous NSCLC, including approximately 17% of patients with previously treated, stable brain metastases. The study demonstrated that pembrolizumab significantly improved overall survival (OS) and progression-free survival (PFS) compared with chemotherapy alone. Importantly, the hazard ratio for OS in patients with brain metastases was 0.41, compared with 0.59 in those without brain metastases, suggesting that ICIs may be beneficial in this subgroup. However, no intracranial-specific response rates or CNS progression data were reported, limiting conclusions about direct ICI activity within brain metastases [95].
- Large Multicenter Cohort Study from France by Descourt et al. [96]: In this study, nearly 20% of patients initiating pembrolizumab for advanced NSCLC had brain metastases. There were no significant differences in response rates, PFS, or OS between patients with and without brain metastases [96].
- Yale Cancer Center Study on Pembrolizumab in Untreated Brain Metastases from Melanoma and NSCLC [24]: In a nonrandomized, open-label, phase 2 trial conducted at Yale Cancer Center, pembrolizumab was administered to 36 patients with at least one untreated or progressive brain metastasis, ranging between 5 and 20 mm in diameter, without associated neurological symptoms. The trial included both melanoma and NSCLC cohorts, with all patients in the NSCLC cohort testing positive for PD-L1 expression. Pembrolizumab achieved a brain metastasis response in 33% of the NSCLC patients, suggesting its potential as an effective systemic immunotherapy for patients with brain metastases from NSCLC [24]. A recently published updated analysis from the same group, involving 65 patients with NSCLC and melanoma with brain metastases, reported that the median time to progression of metastatic lesions in the brain was 5.7–7 weeks, with metastases smaller than 10 mm more likely to show complete resolution [102].
- Retrospective Multicenter Study by Gauvain et al. [98]: This report, by Gauvain et al., included 43 patients with NSCLC and brain metastases, of whom 79% received local treatment to the metastatic lesions. PD-L1 overexpression was not a requirement for inclusion in the study. All patients were treated with nivolumab. The intracerebral activity of nivolumab was similar to its extracerebral efficacy, with a median intracerebral progression-free survival of 3.9 months and a general progression-free survival of 2.8 months. The authors concluded that nivolumab’s intracerebral activity was similar to its reported extracerebral efficacy [98].
- Tsuchiya-Kawano et al. [97] conducted a multicenter single-arm phase 2 trial involving 30 patients with chemotherapy-naïve advanced NSCLC and at least one untreated brain metastases. The patients received nivolumab plus ipilimumab combined with platinum-doublet chemotherapy for two cycles, followed by nivolumab–ipilimumab alone. The study reported an intracranial response rate of 50% (95% CI, 33.2–66.8%) and a complete response rate of 20%. The median intracranial progression-free survival was 8.1 months, underscoring the promising activity of nivolumab and ipilimumab combined with platinum-based chemotherapy in this challenging patient population [97].

### 4.2. Interpretation of Clinical Trial Findings in NSCLC Brain Metastases

Across trials, ICIs have shown promising intracranial activity in select patients with NSCLC brain metastases, particularly those with PD-L1 expression, minimal neurologic symptoms, and no corticosteroid use. However, most large phase III trials have enrolled only small proportions of patients with brain metastases and often lacked intracranial-specific endpoints. Studies that prospectively evaluated untreated brain metastases—such as those by Goldberg et al. [24] and Tsuchiya-Kawano et al. [97]—reported intracranial response rates of 30–50%, suggesting that ICIs can be effective in the CNS when appropriately selected. These results highlight the importance of patient stratification, trial design, and multimodal integration when assessing ICI efficacy in this population.

While existing studies provide encouraging signals of intracranial ICI activity, the current evidence base remains limited and fragmented. Most reports have been retrospective analyses or subgroup observations from larger systemic trials, with heterogeneous inclusion criteria and modest numbers of patients with active brain metastases. Direct intracranial endpoints, such as CNS-specific progression-free survival or response rates, are inconsistently defined.

Moreover, there is a paucity of data integrating correlative biomarkers—such as PD-L1 expression, tumor mutational burden, or immune infiltrates—specifically within brain metastases. The available literature highlights promising efficacy in carefully selected patients but does not yet allow for systematic conclusions regarding patient selection, optimal sequencing with radiation, or long-term CNS control.

These limitations underscore the need for prospective, CNS-focused clinical trials with standardized endpoints and translational correlative studies. Until such data emerge, interpretation of current results should remain cautious, and therapeutic decisions must be individualized within a multimodal framework.

## 5. Challenges and Opportunities in Enhancing the Intracranial Efficacy of ICIs

While ICIs offer promise, several challenges limit their effectiveness in treating brain metastases from NSCLC:

### 5.1. Blood–Brain Barrier Limitations

The blood–brain barrier (BBB) remains a significant obstacle to the effective delivery of immune checkpoint inhibitors (ICIs) and other systemic therapies to brain metastases. Its highly selective permeability restricts the passage of large molecules, including most monoclonal antibodies, thereby limiting therapeutic access to the central nervous system (CNS).

To address this challenge, several strategies are under investigation to transiently disrupt or bypass the BBB and enhance drug delivery:

- Focused Ultrasound (FUS): MRI-guided FUS, in combination with microbubbles, has demonstrated the ability to temporarily and noninvasively open the BBB, facilitating the delivery of therapeutic agents, including ICIs, to targeted brain regions [103,104].
- Bradykinin Analogues: Agents such as labradimil (RMP-7), a bradykinin B2 receptor agonist, have been shown to transiently increase BBB permeability by modulating tight junction integrity, thereby enhancing the delivery of chemotherapeutic agents to brain tumors [105].
- Nanoparticle-Based Delivery Systems: Nanocarriers engineered to exploit receptor-mediated transcytosis pathways have been developed to transport ICIs across the BBB, improving their accumulation within brain tumors [106,107].
- Modulation of BBB Transporters: Strategies targeting endogenous transport mechanisms, such as inhibiting efflux transporters or modulating tight junction proteins, are being explored to enhance the permeability of the BBB and facilitate drug delivery [108].

These approaches aim to overcome the limitations imposed by the BBB, thereby improving the efficacy of ICIs in treating brain metastases. Ongoing research continues to evaluate the safety, specificity, and clinical applicability of these methods.

### 5.2. Heterogeneity of Brain Metastases

Brain metastases in non-small-cell lung cancer (NSCLC) exhibit significant biological heterogeneity, both between patients and within individual tumors [109]. This heterogeneity encompasses variations in tumor antigenicity, immune cell infiltration, mutational burden, and immune checkpoint expression, such as PD-L1. Such diversity contributes to the inconsistent and often unpredictable responses observed with ICIs. For instance, some brain metastases may express high levels of neoantigens and PD-L1, rendering them more responsive to ICIs, while others may remain immune “cold,” lacking T-cell infiltration and exhibiting low immunogenicity, making them more resistant to treatment [79,109].

Moreover, spatial heterogeneity between brain metastases and extracranial lesions poses a unique challenge. Studies have shown discordance in PD-L1 expression and mutational profiles among primary tumors, extracranial metastases, and brain metastases, suggesting that a biopsy from one site may not accurately reflect the immune landscape of another. This discordance complicates treatment decisions and underscores the need for site-specific molecular characterization [110,111].

The tumor microenvironment also varies among brain metastases. Some lesions are surrounded by reactive astrocytes and microglia that can suppress immune activity through cytokine signaling and expression of immunoregulatory molecules, whereas others may be more permissive to T-cell infiltration. These microenvironmental factors can profoundly influence whether or not ICIs will be effective [6].

Given this complexity, personalized treatment approaches are essential. Techniques such as single-cell RNA sequencing, spatial transcriptomics, and radiomic profiling are emerging as tools to dissect this heterogeneity at a granular level. Incorporating these technologies into clinical decision-making may allow clinicians to better stratify patients and tailor immunotherapy strategies, such as combining ICIs with radiotherapy, targeted therapy, or agents that modulate the TME [69,70,112].

Ultimately, addressing the intra- and interlesional heterogeneity of brain metastases is critical for improving therapeutic outcomes in patients with NSCLC who are receiving immunotherapy. Personalized approaches that account for this variability will be key to optimizing the efficacy of ICIs in this challenging patient population [6,113].

### 5.3. Safety and Toxicity Concerns

The administration of ICIs for treating brain metastases in patients with non-small-cell lung cancer (NSCLC) introduces unique safety considerations, particularly related to neurologic immune-related adverse events (n-irAEs). These events, while relatively rare, can be severe and impact patient quality of life and treatment continuity.

#### 5.3.1. Neuroinflammation and Immune-Related Adverse Events

ICIs can disrupt immune tolerance, leading to neuroinflammatory responses within the CNS. Reported n-irAEs include aseptic meningitis, encephalitis, demyelinating disorders, and peripheral neuropathies. The incidence of these events is estimated to be around 1% among patients receiving ICIs, but the risk may be higher in those with pre-existing CNS involvement [114].

#### 5.3.2. Autoimmune Encephalitis

Autoimmune encephalitis (AE) is a serious n-irAE associated with ICI therapy. Clinical manifestations can range from cognitive dysfunction and behavioral changes to seizures and coma. Diagnosis is challenging because of the overlap of symptoms with other CNS pathologies. Management typically involves high-dose corticosteroids, and in refractory cases, treatments such as intravenous immunoglobulin or plasmapheresis may be employed [114,115,116,117,118].

#### 5.3.3. Radiation Necrosis and Edema

In patients who have previously received brain irradiation—such as stereotactic radiosurgery (SRS) or whole-brain radiotherapy (WBRT)—the subsequent introduction of immune checkpoint inhibitors (ICIs) can exacerbate radiation-induced toxicities, notably radiation necrosis and cerebral edema. Radiation causes direct damage to endothelial cells and glia, disrupting the blood–brain barrier (BBB) and triggering hyalinization, demyelination, and vascular injury [119,120]. These changes permit plasma proteins and cytokines to leak into the brain parenchyma, initiating sterile inflammation marked by microglial activation and cytokine release. When paired with ICIs, which boost T-cell activation and promote further immune cell infiltration, this amplified inflammation can compound perivascular damage and hypoxic injury, leading to tissue necrosis. The resulting edema and inflammatory changes often mimic tumor progression on neuroimaging—a phenomenon known as pseudoprogression [121,122,123]. Clinically, patients may experience seizures, focal neurological deficits, or elevated intracranial pressure. Management typically involves high-dose corticosteroids or agents like bevacizumab, which can help reduce inflammation and edema but may also dampen the antitumor immune response. This underscores the need for careful integration of ICI therapy with prior radiation and vigilant monitoring to distinguish between immune-mediated toxicity and true disease progression [124].

#### 5.3.4. Management Strategies

Prompt recognition and management of n-irAEs are crucial. The cornerstone of treatment involves the administration of corticosteroids to mitigate inflammation. Early involvement of neurology specialists is recommended to guide diagnostic evaluation and therapeutic interventions [114,118,125].

#### 5.3.5. Balancing Efficacy and Safety

While ICIs offer promising therapeutic benefits for patients with NSCLC with brain metastases, the potential for serious neurologic toxicities necessitates a careful risk–benefit analysis. Multidisciplinary collaboration among oncology, neurology, and radiology teams is essential to optimize patient outcomes and manage adverse events effectively.

### 5.4. Surgical Resection of Brain Metastases as a Bridge to ICIs

#### 5.4.1. Immediate Benefits and Impact on the Blood–Brain Barrier (BBB):

Surgical resection of brain metastases provides rapid tumor debulking, which can alleviate neurological symptoms, reduce intracranial pressure, and stabilize the patient’s condition [126,127]. This facilitates the early initiation of immune checkpoint inhibitor (ICI) therapy, potentially minimizing delays and decreasing the need for corticosteroids, which are known to suppress immune responses and may blunt the efficacy of ICIs [128,129].

Surgical manipulation inherently disrupts the BBB via direct tissue handling and localized trauma, resulting in a temporary increase in permeability [130,131]. This disruption may allow greater penetration of systemically administered ICIs into the brain parenchyma, potentially enhancing their activity against residual tumor cells or micrometastases [131,132]. The postoperative inflammatory response may further increase BBB permeability and promote immune cell infiltration, potentially amplifying the therapeutic effect of ICIs [133]. However, this enhanced permeability may also carry a risk of increased neurotoxicity, including cerebral edema, immune-related encephalitis, or other inflammatory complications, particularly in patients with pre-existing CNS vulnerabilities [134,135].

#### 5.4.2. Molecular Insights and Personalized Therapy:

Surgical resection also offers the opportunity to obtain tumor tissue directly from the brain metastasis. Molecular profiling of this tissue can reveal actionable mutations, PD-L1 expression levels, tumor mutational burden (TMB), and other biomarkers predictive of ICI responsiveness [136,137]. These insights can support a more personalized approach to systemic therapy, enabling better patient stratification and improved clinical outcomes.

In summary, surgical resection of brain metastases may serve as a valuable bridge to immunotherapy by enhancing drug delivery and enabling molecular characterization. However, the associated risks—particularly those linked to disruption of the BBB and immune-related complications—must be carefully balanced. Multidisciplinary planning and close postoperative monitoring are essential to optimize outcomes when integrating surgery with ICIs.

### 5.5. Stereotactic Radiosurgery (SRS) and ICIs for Treatment of Brain Metastases from NSCLC

For patients with a limited number of brain metastases from NSCLC, stereotactic radiosurgery (SRS) provides targeted, high-dose radiation that can rapidly and effectively control intracranial tumor growth [138,139]. SRS is often used as a bridge to ICI therapy, offering immediate local tumor control while allowing time for systemic immune responses to develop and exert their effects [139]. Notably, SRS and ICIs may exert synergistic effects: radiation can induce immunogenic cell death, releasing tumor-associated antigens that prime the immune system and enhance ICI efficacy, potentially improving both systemic and intracranial tumor responses [139,140,141,142].

In addition, SRS can transiently disrupt the BBB, temporarily increasing its permeability. This disruption may enhance ICI delivery into the CSF and brain parenchyma, albeit in small but potentially clinically meaningful quantities. It may also facilitate immune cell trafficking across the BBB, amplifying immune-mediated antitumor activity. This temporary window of increased permeability presents an opportunity to optimize the timing of ICI administration for greater therapeutic efficacy within the CN [135,143].

## 6. Future Directions and Clinical Implications

Clinical responses to ICIs in brain metastases may differ based on lesion size, number, associated edema, and patient neurological status. Symptomatic lesions, especially if large or causing mass effect, often require urgent intervention such as surgery or radiotherapy before ICIs can be administered. In contrast, small asymptomatic lesions may respond directly to systemic immunotherapy. Clear stratification by symptom burden is essential in future trials to better define patient subgroups who benefit from ICIs alone versus those needing multimodal approaches. To enhance the efficacy of ICIs in treating brain metastases from NSCLC, several strategies are being explored.

### 6.1. Combination Therapies

Combining ICIs with other treatments, such as radiation therapy [144], chemotherapy [145], or targeted therapies [146], may improve intracranial responses by modulating the tumor microenvironment and enhancing immune cell infiltration.

### 6.2. Biomarker Development

Identifying biomarkers that better predict response to ICIs in patients with brain metastases from NSCLC is essential for advancing personalized treatment strategies. Traditional biomarkers such as PD-L1 expression, TMB, and the presence of specific immune cell populations remain under active investigation for their predictive value in the context of the CNS [136,137].

Recent advances in high-throughput technologies, including single-cell RNA sequencing (scRNA-seq), have revealed the molecular and cellular heterogeneity of brain metastases and the intricate landscape of immune infiltration in the central nervous system [68,69,70]. Beyond standard markers, immune gene expression signatures—including interferon-gamma (IFN-γ)-responsive genes [147], CD8^+^ T cell activation markers [148], and T cell exhaustion profiles [149]—may provide predictive insights beyond traditional markers.

In addition, cerebrospinal fluid (CSF)-derived circulating tumor DNA (ctDNA) is emerging as a noninvasive biomarker for intracranial tumor burden and therapeutic monitoring. Compared with plasma, CSF better reflects the molecular profile of CNS tumors due to its proximity to brain metastases and reduced contamination by extracranial disease. CSF ctDNA profiling can track tumor response, detect resistance mutations, and assess clonal evolution in real time. Future integration of CSF analysis into routine ICI monitoring may allow clinicians to stratify patients, detect early progression, and adjust treatment accordingly [150].

### 6.3. Novel Delivery Methods

Developing delivery methods that improve ICI penetration across the BBB, such as nanoparticle-based carriers [106,107] or convection-enhanced delivery [151], could enhance drug concentrations in the CNS and therapeutic outcomes.

## 7. Conclusions

Immune checkpoint inhibitors have significantly advanced the treatment landscape for patients with brain metastases from NSCLC, yet their application in the CNS remains challenging due to the distinct biological and immunological environment of the brain. A deeper understanding of how ICIs interact with tumor cells and the brain microenvironment—including the BBB, immune infiltration, and local inflammation—is essential for optimizing outcomes. Ongoing research into combination strategies (e.g., SRS and ICIs), predictive biomarkers, and innovative delivery methods, among other subjects, holds promise for enhancing therapeutic efficacy and improving survival in patients with brain metastases.

These insights align with recent work specifically focused on metastatic brain tumors, including evidence that astrocytes can act as key immune modulators in the brain tumor microenvironment [65]. Clinical and translational analyses have also evaluated the role of ICIs ± chemotherapy and their use as first-line treatment in NSCLC patients with brain metastases [20,92]. More recently, multimodal genomic and spatial profiling studies have revealed brain-metastasis-specific features such as chromosomal instability, neural-like transcriptional states, and an immunosuppressive microenvironment [152,153]. Together, these findings reinforce the importance of tailoring immunotherapy strategies to the unique biology of brain metastases and highlight the need for CNS-specific endpoints in future clinical trials.

## Figures and Tables

**Table 1:** Summary of clinical trials evaluating ICIs in NSCLC brain metastases.

Trial	ICI Used	Brain Metastases Included	Intracranial Response Rate	Key Findings
CheckMate 017 [93]	Nivolumab	Yes (asymptomatic)	Not separately reported	Demonstrated OS benefit; did not report separate intracranial response data
CheckMate 057 [94]	Nivolumab	Yes (asymptomatic)	Not separately reported	Demonstrated OS benefit; did not report separate intracranial response data
KEYNOTE-189 [95]	Pembrolizumab + chemotherapy	Yes (~17% of patients)	HR for OS in patients with brain metastases: 0.41	HR for OS in brain metastases: 0.41; suggested benefit, but no intracranial response data provided
Yale Phase II (Goldberg et al.) [24]	Pembrolizumab	Yes (untreated, PD-L1+)	33%	Pembrolizumab active in untreated brain metastases
Descourt et al. (French Cohort) [96]	Pembrolizumab	Yes (~20% of patients)	Comparable to nonbrain metastases group	No difference in OS/PFS between brain and nonbrain metastases
Tsuchiya-Kawano et al. [97]	Nivolumab + ipilimumab + chemotherapy	Yes (untreated)	50% (20% CR)	Strong intracranial activity in untreated brain metastases
Gauvain et al. [98]	Nivolumab	Yes (79% received local brain therapy)	Intracranial PFS: 3.9 months	Similar intracranial and extracranial efficacy
Expanded Access Program (Italy) [99]	Nivolumab	Yes (asymptomatic, stable)	DCR 39%, OS 8.6 months	Real-world evidence of ICI efficacy in brain metastases; supported benefit in patients with poor prognoses

Overall survival (OS); hazard ratio (HR); progression-free survival (PFS); disease control rate (DCR).

## Abbreviations

The following abbreviations are used in this manuscript:

NSCLC	non-small-cell lung cancer
SRS	stereotactic radiosurgery
CNS	central nervous system
ICIs	immune checkpoint inhibitors
BBB	blood–brain barrier
VEGF	vascular endothelial growth factor
MMPs	matrix metalloproteinases
RT	radiation therapy
WBRT	whole-brain radiation therapy
n-irAEs	neurologic immune-related adverse events
OS	overall survival
ctDNA	circulating tumor DNA
EMEC	early metastatic epithelial cell clusters
